# Menopause and Sustainable Career Outcomes: A Science Mapping Approach

**DOI:** 10.3390/ijerph182312559

**Published:** 2021-11-29

**Authors:** Beatrice I. J. M. Van der Heijden, Karen Pak, Mónica Santana

**Affiliations:** 1 Institute for Management Research, Radboud University, 6500 HK Nijmegen, The Netherlands; karen.pak@ru.nl; 2 Faculty of Management, Open Universiteit, 6419 AT Heerlen, The Netherlands; 3 Department of Marketing, Innovation and Organisation, Ghent University, 9000 Ghent, Belgium; 4 Business School, Hubei University, Wuhan 430062, China; 5 Kingston Business School, Kingston University, Kingston upon Thames, London KT2 7LB, UK; 6 Management and Marketing Department, University of Pablo de Olavide, Carretera de Utrera Km. 1, 41013 Seville, Spain; msanher@upo.es

**Keywords:** menopause, workplace, sustainable career perspective, science mapping approach

## Abstract

This paper provides a systematic review of the phenomenon of menopause at the workplace from a sustainable career perspective, by highlighting its major themes along with the evolution and tendencies observed in this field. A conceptual science mapping analysis based on co-word bibliographic networks was developed, using the SciMAT tool. From 1992 to 2020, 185 documents were retrieved from the Web of Science. In the first analyzed time span (1992–2002), *postmenopausal women*, *health*, and *risk factors* appeared to be the motor themes (well-developed and important for the structure of the discipline under focus), and *disorder* was an emerging or disappearing theme in the phenomenon under research. In the second studied period (2003–2013), *risk* and *health* were motor themes, *menopausal symptoms* was a basic or transversal theme (important for the discipline but not well-developed), *coronary heart disease* was a specialized theme (well-developed but less important for the structure of the research field), and *postmenopausal women* was an emerging or disappearing theme (both weakly developed and marginal to the field). In the third studied period (2014–2020), *menopause*, *breast cancer*, and *menopausal symptoms* were motor themes, *Anxiety* was a specialized theme and *risk* and *body mass index* were emerging or disappearing themes. Sustainability of women’s careers in the second half of life is of increasing importance given the increasing equal representation of men and women in working organizations, and the impact of the changing nature of work in the 21st century on older workers.

## 1. Introduction

The concept of menopause has been tackled extensively in the scientific literature, and it has resulted in a large number of publications in medical sciences publications (see for example [1]). Menopause refers to the point in a woman’s life when she has not menstruated for a year [2] (p. 7), which usually occurs around the age of 50 [3]. The transition from the time that the first-time menopausal symptoms begin and the post-menopausal stage can last for over a decade. Although menopause is normally a natural phenomenon, some women may experience menopausal symptoms at an earlier age due to medical treatment [2]. In the context of work, previous research already showed that menopausal symptoms may cause depression and anxiety among female employees, however these complaints vary greatly between women (see [4] for an overview). Moreover, menopausal symptoms evoke many stereotypes and meta-stereotypes (see for example [5,6]) which makes it difficult for female employees who experience (post-) menopausal symptoms to discuss this in the workplace [7,8]. 

Notwithstanding the importance of this highly complex phenomenon in the workplace [4,9], given the dynamics related to it, there is a serious lack of research on the phenomenon of menopause at the workplace, let alone taking into account a sustainable career perspective [10,11]. Sustainable careers are defined as “sequences of career experiences reflected through a variety of patterns of continuity over time, thereby crossing several spaces, characterized by individual agency, herewith providing meaning to the individual” [12] (p. 7). 

With the increasing equal representation of men and women at the workplace [13], it is distressing that there is still a serious inattention to the impact of menopause at the workplace, which is likely due to the taboo surrounding the topic [4,14]. Departing from a sustainable career perspective [10,11], we argue that experiences related to menopause might have consequences for women’s careers because bodily changes and hormonal fluctuations [15,16] might intersect with their perceived health (e.g., work ability, burn-out), happiness (e.g., job satisfaction, motivation to continue working) and productivity (e.g., job performance, employability) which are the core indicators of sustainable careers [10,11,17], as well as with the experiences important key figures (e.g., one’s supervisor, colleagues, family and friends) in their environment might have. The latter might be translated into negative stereotypes about menopausal women [18,19]. Given the hormonal changes and the stereotyping that occurs during menopause, we argue that it is likely that the career sustainability of women is affected during the menopause. 

In the exemplary work by Jack and colleagues [20], it was reported that in some studies employed women reported fewer and/or less severe, intense or burdensome physiological and symptoms and somatic complaints, in comparison with unemployed women. In a similar vein, they found that psychological symptoms, such as anxiety disorders were more often reported by unemployed women. By contrast, other research has failed to establish a link between symptoms and employment status (see [14] for all specific references). In a similar vein, these scholars also found that managerial status and occupation may make a difference to symptom reporting, with non-managerial women suffering relatively more compared to women in managerial positions. Interestingly, Jack and colleagues [20] also found that occupational classification may affect the menopausal onset, with white-collar workers experiencing it later than blue-collar workers, although this effect was disputed in some other studies as well. Seen from a wider economic perspective, it is important to report Mvundura’s finding that menopause creates an increased supply of women in the US labor market, and that perimenopausal women are more likely to be self-employed in comparison with postmenopausal, and that early menopause was associated with self-employment as well [21].

Given the supposed linkage between menopause and women’s career sustainability, this paper aims, firstly, to investigate the phenomenon of menopause at the workplace in the scholarly literature, from a sustainable career perspective, published until now, by conducting a bibliometric analysis, and, secondly, to highlight its major themes along with the evolution and tendencies observed in this field of interest. In the scholarly work by Grandey et al. [4], a clear overview of the hormonal and physiological changes was provided. To avoid overlap with this previous research in the field, we have decided not to focus on the medical side of menopause in our review but to emphasize the relationship between menopause and sustainable career outcomes. However, given the centrality of the medical literature stream a succinct summary is given here. As described by Grandey and associates [4], menopause involves hormonal changes, such as reduced estrogen paired with increased follicle-stimulating hormone, which leads to testosterone becoming more dominant [22]. These hormonal changes are portrayed by means of physiological indicators such as unpredictable hot flashes and night sweats (i.e., vasomotor symptoms) and cycle length variability, amenorrhea, and heavier or lighter flow than usual (ibid.). For some women, the hormonal changes related to menopause can even result in depression [23], anxiety and distress [4] which may interfere with their performance at work as well [7]. 

As indicated above, our research focuses on the phenomenon of menopause at the workplace from a sustainable career perspective. Specifically, this study uses a science mapping analysis [24], being a type of bibliometric research methodology [25,26]. Despite previous reviews on menopause at the workplace that use traditional methods [2,4], this work presents a type of bibliometric analysis which allows to offer a graphical visualization of this field, identifying the most important themes and its evolution. To conduct this science mapping analysis, this study employs the SciMAT tool [27], carrying out a co-word analysis that builds strategic graphs that classify the detected themes and its evolution. Specifically, various research themes and their evolution were offered for three differentiated periods 1992–2002, 2003–2013, and 2014–2020, highlighting the main research contribution in each period. By offering a critical evaluation of the outcomes of this science mapping research, we aim to obtain thorough insight into the highest impact research strands so far in the domain of menopause at the workplace from a sustainable career perspective, which will enable us to come up with promising research questions for future endeavors in this field in order to fill gaps that we distill from our review approach. The outcomes of our work can help to determine what specific research questions and empirical work can be done to move the highly needed research in this field forward. 

After this introduction, we will go into our underlying theoretical framework pertaining to a sustainable career perspective on the phenomenon of menopause at the workplace. Next, we will go into the methodological approach to analyze the literature in this domain, through a science mapping study. The subsequent section will go into the findings of our bibliometric review, followed by a discussion section wherein we reflect on our outcomes and propose recommendations for future scientific work in this field. 

## 2. Theoretical Framework

A sustainable career perspective implies that careers are approached from the *individual perspective*, being the central career actor, in our case the menopausal woman. However, by incorporating a *multiple-stakeholder perspective* (cf. [28]) as well, situational constraints and opportunities within the individual’s context that affect their attitudes and behaviors [29], and stakeholders, such as their family, friends and peers, supervisor, colleagues and employer, operating within this context, need to be taken into account as well. For example, support from family, friends and colleagues could help menopausal women to better deal with their symptoms, whereas stereotyping, expressed by means of ridicules from co-workers, concerning symptoms such as hot flashes, or even discrimination by the employer, might hinder the sustainability of their career. In addition, sustainable careers comprise a *dynamic process*, as both factors related to the person and their context change over time, thereby affecting the sustainability of their career [10]. 

Following this line of reasoning, it is inherent to any individual’s career that events and evolutions in the person, in our case the phenomenon of menopause at the workplace, and factors related to this in their context, affect a woman’s experiences and may bring along opportunities as well as hindrances and challenges to deal with. Analogously with De Vos et al. [10], we posit that it is how women and the other stakeholders involved deal with those personal and contextual changes related to menopause might affect the career sustainability of women over time. Therefore, it is of utmost importance to shed more light on the phenomenon of menopause at the workplace from a sustainable career perspective, not in the least place as women, for social responsibility, demographic, legal and business reasons, should be supported to work at least until their retirement age, by developing and maintaining inclusive workplaces [2].

A sustainable career perspective on the topic of menopause is novel as it opens up this scholarly field by proposing a commitment to move scholarly research from a micro-level or bio-medical attention (e.g., [30]) towards a meso-level or workplace context and attitudes approach (see [2] who differentiate between micro-, meso-, and macro-levels, that is structural and cultural issues dealing with the relationships between individuals, the state, the economy and wider society) for more information on the different levels of attention for the phenomenon of menopause, and preferably, even towards a whole-life perspective [11,31]. In doing so, we aim to develop insights that might help women and their surrounding stakeholders to adequately deal with the phenomenon, and to protect and preferably further enhance women’s career sustainability across the life-span. In order to move the field forward, we first need to systematically investigate the ‘state-of-the-art’ of the research in the field, determining its knowledge gaps and, through this, identify a future research agenda. 

## 3. Method

The goal of this paper is to provide a bibliometric analysis of the phenomenon menopause at the workplace from a sustainable career perspective. Bibliometrics was chosen, instead of a meta-analytic approach, as it allows for a wider scope. A meta-analytical search for integrated empirical evidence from quantitative research in a certain field is a relevant technique, but inherently limited in the type and breadth of the included studies ([26], p. 436; [32]), while the bibliometrics method can study any type of research and relationships in a certain field, with immature fields, such as the phenomenon of menopause at the workplace, being no exception. To this end, we conducted a science mapping analysis, an important part of the bibliometric analysis [25]. Bibliometrics refers to the methodology that studies texts and information in a statistical and mathematical manner [33,34] and thus bibliometrics introduces a measure of objectivity into the evaluation of academic literature [35]. This is considered a robust statistical method to derive conclusions regarding the findings from previous research [36]. In this paper, we build on science mapping analysis to derive a visual representation that shows how the themes, articles and authors of the identified studies are interrelated [24]. Science mapping targets to show the structure and dynamics of a field of study [26]. SciMat [27] was used to conduct this analysis, as it combines science mapping with performance analysis techniques. This has enabled us to study and visualize the research on the phenomenon of menopause at the workplace from a sustainable career perspective, and to identify the thematic evolution of this line of research. Although there are several science mapping techniques such as VOS viewer, Biblioshiny or Bibliometrix [37], this study used SciMAT due to its strong pre-processing and export characteristics. Particularly, SciMAT enables to analyze the evolution of a discipline, to conduct a keywords normalization process, and to enrich the classification of research themes with performance measures (citations, documents, etc.)

In line with Börner et al. [38] and Cobo et al. [25], we conducted the following steps in our science mapping analysis: (1) data search; (2) data refinement; (3) creation of the network and normalization; (4) map creation; (5) analysis and visualization; and (6) performance analysis. In the first step (i.e., data search), we searched in the Web of Science (WoS) database (www.webofknowledge.com (accessed on 16 December 2020) for relevant articles (see Table 1 for the string that we used). As our study targets the analysis of the impact of menopause from a sustainable career perspective, considering a multidisciplinary approach, the Web of Science database was selected. We decided not to select the PubMed database as it has a strong bias towards medicine and biomedical sciences, which does not fit with the goal of our study, while the Web of Science represents the most complete content coverage in the fields of science, social sciences, arts, and humanities [39,40]. 

We searched in the following research areas given the interdisciplinary character of the phenomenon of menopause at the workplace: Public Environmental Occupational Health, Behavioral Sciences, Business, Management, Psychology Applied, Ergonomics, Psychology, Psychology Multidisciplinary, and Social Sciences Interdisciplinary, as they offer a wide overview of our target research theme. This WoS search resulted in 185 articles on the phenomenon of menopause at the workplace from a sustainable career perspective up to 2020. All identified papers were published in the period between 1992 and 2020. We were not able to identify any research on this topic prior to 1992. In this study, a conservative approach was followed by not reducing the number of obtained articles. These 185 articles were scanned and all appeared to be related to the topic. 

In the second step (i.e., data refinement), we refined and reduced the data. We scanned the keywords to identify any word that was incorrect, duplicate or misspelled. For instance, words such as human resource management or HR were joined because they represent the same concept. In the third step (i.e., creation of the network and normalization), we opted for the co-occurrence choice to obtain the networks, and for the equivalence index to normalize the network. In the fourth step (i.e., map creation), the simple centers algorithm was used to identify clusters or themes. In the fifth step (i.e., analysis and visualization), strategic diagrams, clusters and evolution maps were offered. In this stage, we first identified research themes using co-word analysis [41]. According to Callon et al. [41], a co-word analysis is a content analysis method that employs the words in documents to comprehend relationships and to obtain networks of the themes of a scientific discipline. In this case, the unit of analysis is a certain concept, not a document, journal or author (ibid.). Next, we clustered keywords around topics and themes [42] in order to detect keyword networks and corresponding research problems. Then, we visualized these networks using strategic diagrams and thematic networks [25]. These diagrams and networks were based on two characteristics, namely centrality and density [43]. The concept of centrality refers to the amount of interaction of one network with other works. This is an indicator of the importance of this theme in the development of the research field under study. The term density alludes to the internal strength of the network and reflects the development of this theme. Based on these two characteristics, we can group themes into three categories: (1) motor themes (high in both centrality and density); (2) specialized themes (low in centrality, but high in density), (3) emerging or disappearing themes (low in both centrality and density), and (4) basic themes (high in centrality, but low in density). 

Subsequently, thematic areas were identified. For each time period, the key research themes were detected and analyzed to give an overview of the way the field evolved over the years. This was visualized in a so-called evolution map [25]. Finally, a performance analysis was conducted. In this final phase, the relative contribution of all research topics was measured against the entire knowledge field in order to identify the most important subfields. 

## 4. Performance Analysis of the Bibliometric Data

In this section, we report on our analyses of the literature on the phenomenon of menopause at the workplace from a sustainable career perspective through the study of the following bibliometric indicators: published articles, citations received, most productive authors, most cited publications and most prolific journals.

### 4.1. Published Articles and Citations Received

**Figure 1:**
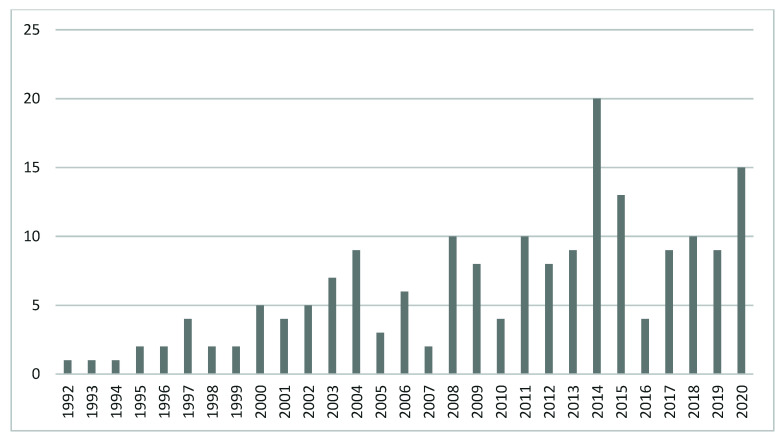
Number of WoS publications on the phenomenon of menopause at the workplace from a sustainable career perspective, from 1992 to 2020.

**Figure 2:**
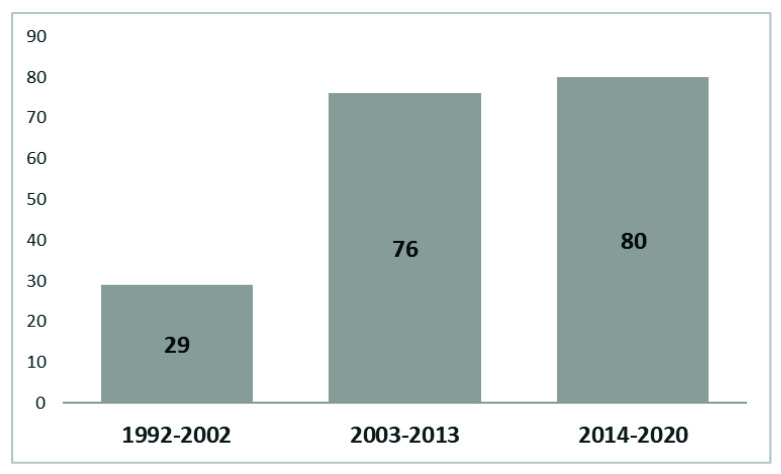
International Web of Science (WoS) publications on the phenomenon of menopause from a sustainable career perspective.

Figure 1 offers the yearly number of publications related to the phenomenon of menopause at the workplace from a sustainable career perspective, being our field of study, showing its growing trend. During the first 10 years, there was a small upward trend, reaching the amount of 5 publications in the year 2003, which was never seen before. In the year 2014, for the first time, the number of publications in the field reached the level of 20 documents. Therefore, the initial periods of research in this domain were separated into two equal parts: 1992–2002 and 2003–2013. The time span from 2014 to 2020 appeared to be the most productive for the scientific domain of menopause at the workplace from a sustainable career perspective. During 1992–2002, 2003–2013, and 2014–2020, the number of published articles and reviews were 29, 76 and 80, respectively (see Figure 2).

**Figure 3:**
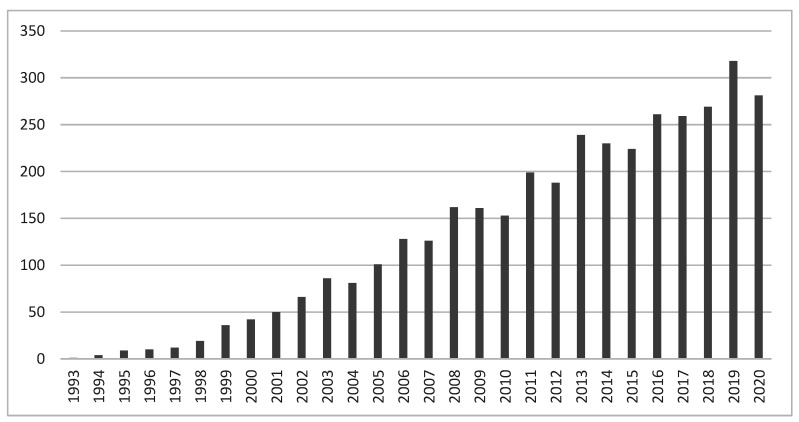
Number of WoS citations on the phenomenon of menopause from a sustainable career perspective, from 1992 to 2020.

Figure 3 indicates the number of citations of publications on the topic of menopause at the workplace from a sustainable career perspective for each year. The distribution of the citations shows a positive trend, surpassing the number of 200 citations, for the first time, in the year 2013, and the number of 300 citations in the year 2019. From 1992 to 2020, a total of 3716 citations were recorded. In addition, according to the WoS, across this time period, the average citation rate per document was 20.09, thus, a positive tendency can be expected over the years to come.

### 4.2. Most Productive Authors, Most Cited Publications and Most Prolific Journals

To shed further light on this scientific domain, we detected the most productive authors, cited documents and journals. Table 2 shows the authors with 2–4 documents, with the most prolific authors being Raczkiewicz and Bojar, with 4 publications each, followed by their peers (i.e., Kingsberg, Simon, Hirokawa, Humeniuk, Grochans, etc.), with each having produced 2 publications in this domain in the WoS.

Documents with more than 50 citations in the WoS are presented in Table 3. The most cited publication is the one by Gold et al. [44] entitled “Factors associated with age at natural menopause in a multi-ethnic sample of midlife women”, with 488 citations since 2001. One of the most recent publications with the highest number of citations is the systematic review by Duijts and associates [45] named “Physical and psychosocial problems in cancer survivors beyond return to work: a systematic review”, with 139 citations since 2014. As early or accelerated menopause can be a consequence of cancer treatment, this work is not to be ignored. 

The most prolific journals in this scholarly domain, with at least 4 publications, are presented in Table 4. BMC Women’s Health, International Journal of Environmental Research and Public Health, and Journal of Women’s Health are among the journals with the highest number of publications on the topic of menopause at the workplace from a sustainable career perspective (10 publications each). As shown in Table 4, there is a variety of journals with publications on the topic, which highlights the growing interest from well-reputed journals in this scientific field.

## 5. Science Mapping Analysis

### 5.1. Analyzing the Evolution of the Specific Research Themes for the Phenomenon of Menopause at the Workplace from a Sustainable Career Perspective

#### Evolution of the Scientific Domain

**Figure 4:**
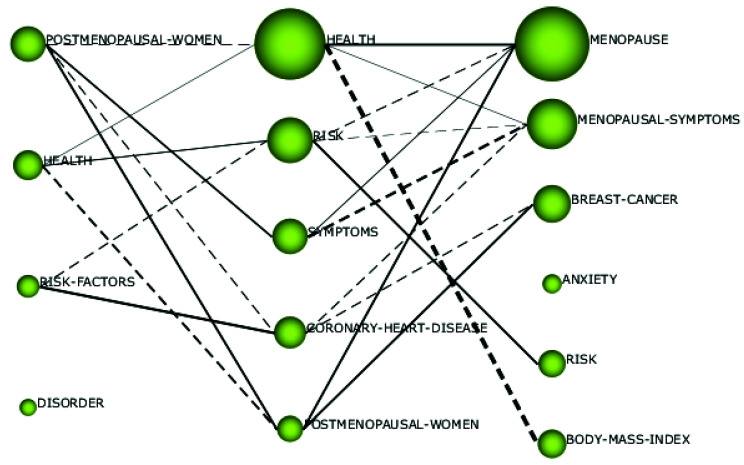
Evolution map of the link between menopause and work’s themes for periods 1992–2002, 2003–2013, and 2014–2020.

As can be seen in Figure 2, only 29 articles regarding the phenomenon of menopause at the workplace from a sustainable career perspective were published in the period from 1992 to 2002. From 2003 to 2013, the research interest in this domain increased, and 76 articles on the phenomenon came out. From 2014 to 2020, 80 additional articles were published. In addition, Figure 4 gives us more insight into how research regarding the phenomenon of menopause at the workplace from a sustainable career perspective evolved over the years. The size of the spheres in Figure 4 indicates the importance of each topic in the different time periods.

From 1992 to 2002, the focus was on the topics *postmenopausal women*, *health*, *risk factors*, and *disorder*. From 2003 to 2013, the topic of *postmenopausal women* continued to attract attention, but lost some of its importance, and was partly incorporated in research on *symptoms,* whereas the topic of *women* was incorporated in research on *health*, *symptoms*, and *coronary heart disease*. The topic of *risk factors* appeared to continue under the topics *risk* and *coronary heart disease*. Moreover, during this time period, research on *health* gained importance and became the most influential topic. 

From 2014 to 2020, the topic of *health* was incorporated in the topic of *menopause*, which was the most influential topic of this period, and the topic of *body-mass index* was also incorporated. *Symptoms* evolved into *menopausal symptoms*, and the topic of *postmenopausal women* was incorporated under *menopause* and *breast cancer*. The topic *coronary heart disease* continued under the topic of *breast cancer*, while the topic of *anxiety* emerged as a new research theme in this time period. We will analyze each sub-period in more detail below.

### 5.2. Analysis of the Scientific Domain in Each Sub-Period

#### 5.2.1. Sub-Period 1992–2002

**Figure 5:**
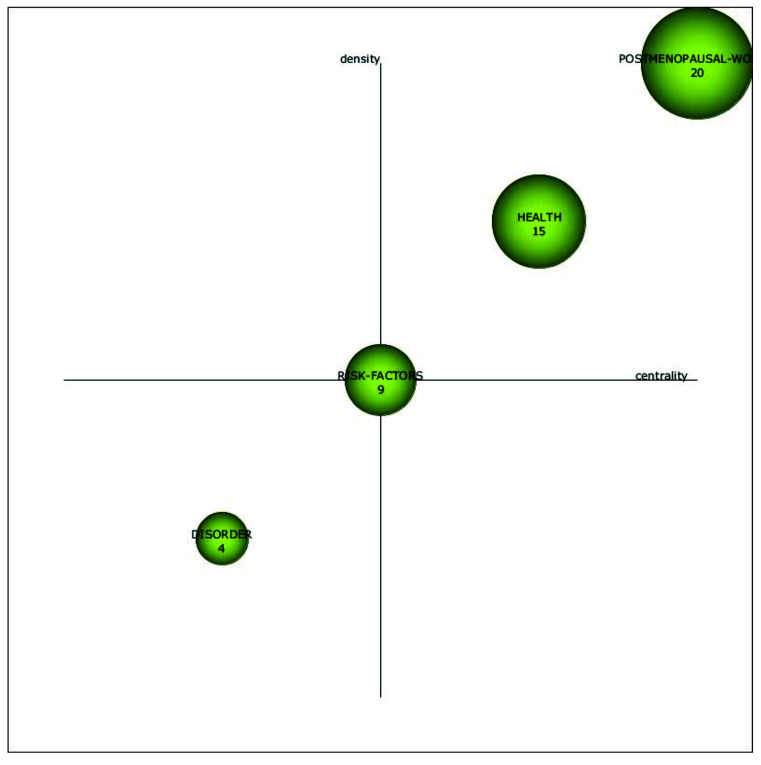
Strategic diagram of the link between menopause and work’s themes from 1992–2002. (Number of documents).

As can be seen in Figure 5, the topics of *postmenopausal women*, *health,* and *risk factors* were motor themes (well-developed and important for the research field) in the sub-period 1992–2002, whereas *disorder* was an emerging theme. Within the motor theme *postmenopausal women*, employment status was often used as an antecedent of risk factors for menopausal women (see for example [44,46]). In several other studies, work was used as a contextual variable to better understand narratives on menopausal experiences (see for example [47,48,49]. In particular, Hunter et al. [49] found that contextual variables, such as employment status, influenced the degree to which postmenopausal symptoms were considered as troublesome. Unfortunately, most of these studies only looked at whether a person was employed or unemployed but did not take specific job characteristics into account. An exception to this was the study by Davis et al. [50], who found that employed women, and especially those with high levels of stress and low levels of supervisor support, had elevated levels of fibrinogen regardless of their menopausal status.

With regards to the motor themes *health* and *risk factors*, as well as the emerging theme *disorder*, in the sub-period 1992–2002 papers focused, amongst others, on the influence of breast cancer history on age of natural menopause ([44]; no effect found), menopause and work as separate predictors of a variety of health complaints such as coronary heart disease, drug use, and breast cancer [51], the risk of breast cancer as an important factor in the decision of undergoing hormone replacement therapy to lighten consequences of post-menopausal symptoms (with employment status as another factor in the decision-making process) [49], and occupations with heightened risk for breast cancer before and after menopause [52]. 

#### 5.2.2. Sub-Period 2003–2013

**Figure 6:**
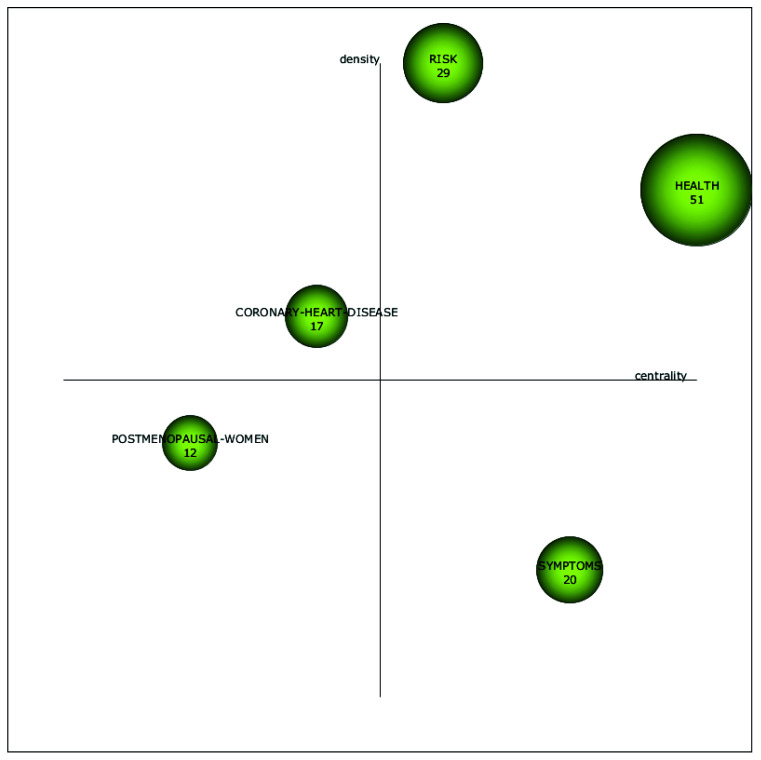
Strategic diagram of the link between menopause and work’s themes from 2003–2013. (Number of documents).

In the sub-period 2003–2013, the motor themes were *risk* and *health* (see Figure 6). *Symptoms* was a basic theme (important for the field but not well-developed), whereas *coronary heart disease* was a specialized theme (well-developed but less important for the field). The topic of *postmenopausal women* was a disappearing theme (weakly developed and marginal to the field). With regards to the motor theme *risk*, Kleinman et al. [53] found that women with diagnosed postmenopausal symptoms had a higher amount of sick leave days, higher sick leave costs and lower productivity compared to women of the same age who were not diagnosed with postmenopausal symptoms. Furthermore, Popovic et al. [54] found that occupational exposure to lead was associated with a younger menopausal age. Moreover, employment and menopausal status were used as two separate outcome variables of breast cancer treatment [55] and as predictors of mental health status [56]. 

With regards to the motor theme *health*, Cassou et al. [57] found that facing menopause at an early age was associated with high-strain jobs and difficult work schedules, whereas having menopause at a later age was associated with a higher educational background and repetitive work. This suggests that job characteristics may indeed influence the timing of one’s menopause. Furthermore, Fallahzadeh [58] found that employed postmenopausal women reported a better quality of life compared to unemployed postmenopausal women. In addition, Sasser et al. [59] indicated that postmenopausal women who develop chronic conditions during their menopause have higher odds for indirect work-related costs, for example due to job loss. As far as the basic theme *symptoms* is concerned, there appears to be a lot of overlap with the motor theme *health*. Articles in this sub-theme discussed the influence of menopausal symptoms on work outcomes, and the influence that job characteristics have on menopausal symptoms as discussed above. 

With regards to the specialized theme *coronary heart disease*, studies found that there were several risk factors at work that could cause coronary heart disease along with severe menopausal symptoms, especially among menopausal women (see for example [60,61].

#### 5.2.3. Sub-Period 2014–2020

**Figure 7:**
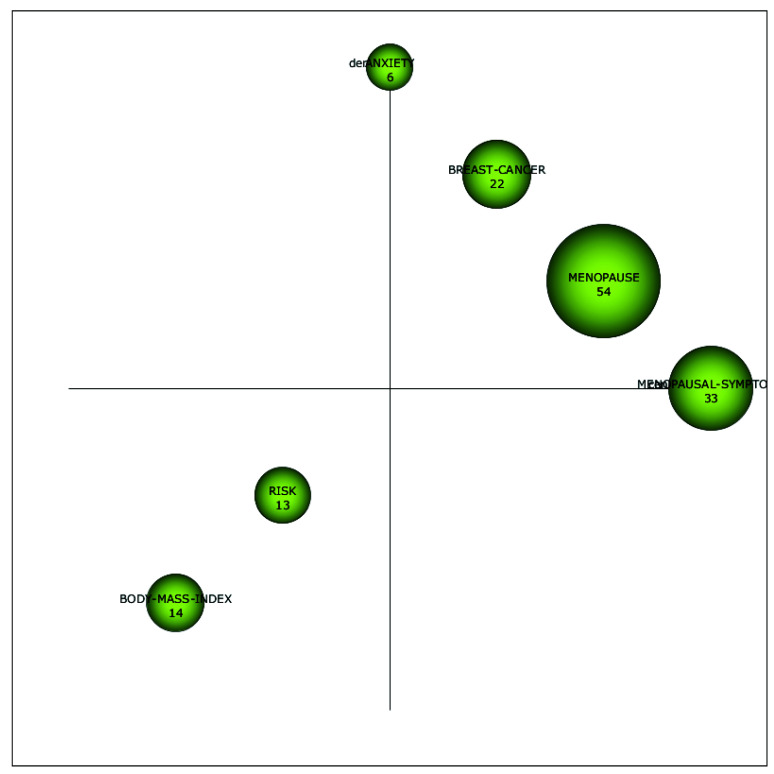
Strategic diagram of the link between menopause and work’s themes from 2014–2020. (Number of documents).

As can be seen in Figure 7, *menopause*, *breast cancer*, and *menopausal symptoms* were motor themes (well-developed and important for the discipline) in the period from 2014 to 2020. *Anxiety* was a specialized theme (well-developed but less important for the research field) and *risk* and *body mass index* were emerging themes (both weakly developed and marginal to the field).

**Figure 8:**
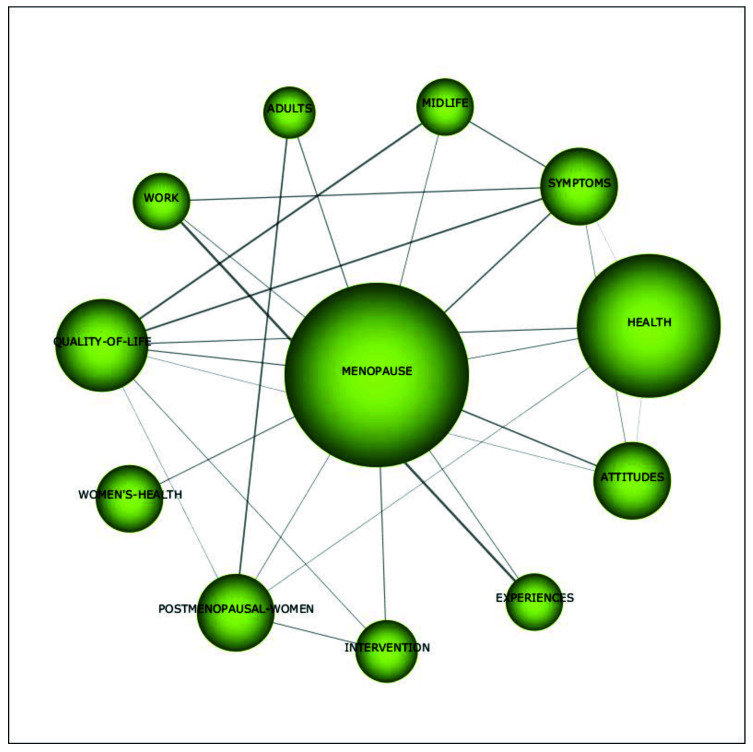
Cluster’s network of the link between menopause and work’s themes (menopause) for the period 2014–2020.

**Figure 9:**
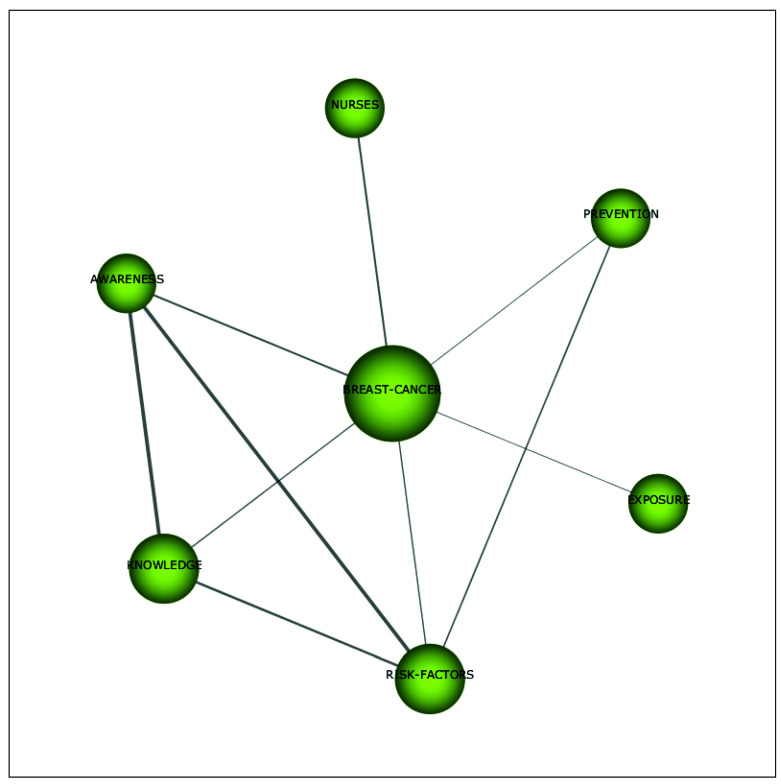
Cluster’s network of the link between menopause and work’s themes (breast cancer) for the period 2014–2020.

Figure 8 shows that the motor theme *menopause* covers topics such as *health, postmenopausal women*, *symptoms*, *work*, *intervention*, *experiences*, *adults*, *quality of life*, *attitudes*, and *midlife*. In the period of 1992 to 2002, in the majority of studies within the motor theme *postmenopausal women*, employment was often used either as an antecedent of risk factors or as a contextual variable to better understand narratives on menopausal experiences. Although in the period of 2014 to 2020, there were still a few studies that examined the phenomenon of menopause at the workplace from a sustainable career perspective in a similar way (see for example [62,63], generally the field evolved, and now scholars researched this domain in a variety of more advanced ways. For example, Grandey and colleagues [4] and Atkinson and associates [2] reviewed the literature on the phenomenon of menopause at the workplace and made recommendations for future research. Other studies examined the impact of menopausal symptoms on a variety of sustainable career-related outcomes, such as work ability and burn-out (see for example [64,65]). Moreover, several interventions aimed at improving work ability and quality of life during menopause were performed (see for example [66,67]). Finally, the study of Steffan [9] described how women dealt with menopausal symptoms at work. As regards the second motor theme, i.e., *breast cancer*, from 2014 to 2020, the topics of *prevention*, *awareness*, *knowledge*, *nurses*, *risk factors*, and *exposure* were covered (see Figure 9). These studies mainly discussed how awareness and knowledge about breast cancer could be increased, and which risk factors could lead to breast cancer. Moreover, Duijts et al. [45] found that cancer survivors experienced ongoing physical and psychological problems, such as menopausal symptoms, which can cause difficulties at work. 

**Figure 10:**
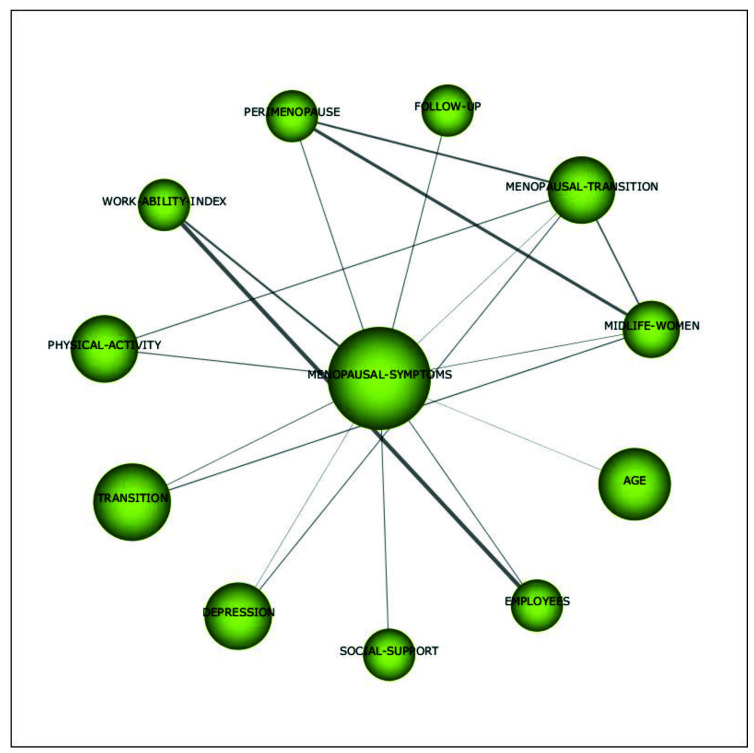
Cluster’s network of the link between menopause and work’s themes (menopausal symptoms) for the period 2014–2020.

Within the motor theme *postmenopausal symptoms*, topics such as *work ability*, *depression*, *social support*, and *physical activity* were discussed (see Figure 10). For example, Wieder-Huszla et al. [68] found that age, education, and employment status have significant effects on the quality of life after the menopause. Moreover, Abdelrahman and colleagues [69] found that working women are more likely to have higher stress levels compared to women who do not work, while higher educational levels appeared to be associated with lower levels of stress. Additionally, Watkins et al. [70] reported that several female fire fighters experienced problems at work due to menopause.

Within the specialized theme *anxiety*, depressive symptoms during menopause were often discussed. For example, Grochans et al. [71] found that employment during menopause can reduce the likelihood of depressive symptoms. Finally, as regards the first emerging subtheme, i.e., *body mass index*, most studies discussed weight and employment status as separate indicators for age at menopause or menopausal symptoms. Within the second emerging subtheme, i.e., *risk*, studies discussed risk factors for early menopausal symptoms (such as having a hysterectomy, smoking, and occupational exposure to lead). In the next section, we will go into a reflection upon the outcomes of our scholarly work, followed by a discussion of its limitations and recommendations for future research aiming to help close the gap in this scientific domain.

## 6. Conclusions, Limitations and Future Research Agenda

### 6.1. Reflecting upon the Outcomes

In this scholarly work, a bibliometric analysis, using the SciMAT tool, was performed; it allowed us to gain deep knowledge about the most prolific themes related to the research topic of the phenomenon of menopause at the workplace from a sustainable career perspective. By conducting a critical evaluation of the existing literature and providing insight into the research strands that have the highest impact until now, we aimed to determine the maturity of the field and to come up with suggestions for further development of the topic. From our bibliometric analysis, we can conclude that there is a serious lack of research on the phenomenon of menopause at the workplace, let alone focusing upon how a female’s career sustainability is influenced by this phenomenon. The mapping of results, as reported in the section entitled ‘Performance Analysis of the Bibliometric Data’ above and the accompanying figures that are portrayed there, clearly show what research endeavors were undertaken up until now, and where future research projects can add to the existing scholarly knowledge. 

In particular, we were able to retrieve 185 documents from the WoS, spanning the time period from 1992 to 2020. It is striking that we were not able to find any research on the phenomenon of menopause at the workplace from a sustainable career perspective before 1992. The analysis of the evolution of the specific research themes, that we have found, indicates that in the first analyzed time span (1992–2002), *postmenopausal women*, *health*, and *risk factors* appeared to be well-developed themes and important for the structure of the discipline (i.e., motor themes), and that *disorder* was an emerging or disappearing theme. In the second studied period (2003–2013), *risk* and *health* came up as motor themes, *menopausal symptoms* appeared to be important for the discipline but not well-developed (i.e., a basic or transversal theme), *coronary heart disease* was well-developed but less important for the structure of the research field (i.e., a specialized theme), and *postmenopausal women* was both weakly developed and marginal to the field (i.e., an emerging or disappearing theme). In the third studied period (2014–2020), *menopause*, *breast cancer*, and *menopausal symptoms* appeared to be motor themes, *anxiety* was a specialized theme, and *risk* and *body mass index* were emerging or disappearing themes.

From our findings, we may conclude that the literature on the phenomenon of menopause has focused strongly on the micro-level or bio-medical aspects related to menopause (e.g., [30]), and that the literature, just very recently, portrays an evolution into a meso-level or workplace approach [2]. Regrettably, so far, a whole-life perspective [11] on the impact of menopause on women’s career sustainability is completely absent in the scientific literature. We argue that a whole-life perspective, wherein the work-nonwork interface in career development [31] (see also [72,73]) is incorporated, and wherein societal, organizational and psychological concerns related to this interface are dealt with, are needed to do justice to the complexity of the phenomenon [4,9]. Adopting such a whole-life perspective would also imply that stakeholders that are surrounding the individual career holder (i.e., their family, friends and peers, supervisor, colleagues and employer) are taken into account [11]. 

### 6.2. Limitations of This Study and Recommendations for Future Research

We hope that the systematization and representation of the field of menopause at the workplace from a sustainable career perspective, that has been set out in this paper, will foster new research avenues. To start with, menopausal experiences are highly idiosyncratic and inconsistent across time, because of fluctuating levels of hormones and different contexts women move through, both in their private and work life. Therefore, we posit that the three dimensions of person, context, and time [10,11], that form the basis of the notion of sustainable careers, should be studied in tandem. So far, the majority of research has focused on biomedical processes that mostly neglect the (work) context. An interesting path for more empirical work could be to examine how hormonal fluctuations interact with individual coping strategies and with support offered by the woman’s partner, employer, supervisor or colleagues, and how this, in turn, affects their health, happiness, and productivity over time. Related to this, future research could focus on which actions the partner, employer, supervisor and colleagues could undertake to support women who experience menopausal symptoms in maintaining their health, happiness, and productivity during the menopausal transition. 

In a similar vein, future work should not consider the three distinguished sustainable career indicators (i.e., health, happiness, and productivity) [10,11,17] in isolation. Rather, we recommend future researchers to combine outcomes such as work ability (i.e., health), engagement (i.e., happiness), and performance (i.e., productivity), to better understand possible interactions over time. More scholarly work is also needed to examine the role of personal factors, such as one’s personality, capabilities and competencies, on the one hand, and contextual factors, such as one’s private life, one’s social support systems, available job resources/job design, and HRM practices at the workplace, on the other hand, in coping with menopause at the workplace, and how this coping affects one’s career sustainability. 

We also call for more research pertaining to different categories of menopausal women, such as those working in regular organization-based employment, temporarily employed or contingent workers, self-employed women, as well as independent workers operating outside working organizations and in established professions [74]. It is not unlikely that the dynamic process of dealing with menopause at the workplace differs across these categories of workers, given the variety in the social support systems and HRM policies and practices these categories of women may count on. In addition, comparative cross-country research is valuable in order to determine whether differences in institutional contexts, such as policies regarding work-life balance, and the availability of age-related tailor-made HRM practices (see also [75,76]) might have an effect on the career sustainability of menopausal women.

More empirical work, using person-centered approaches, is also needed to better understand intra-individual change trajectories over time (cf. [77,78]) (e.g., related to changes in one’s personal or work-related context, changes in one’s occupational or societal context) of women going through menopause, as well as inter-individual changes over time (e.g., different categories of menopausal workers, as outlined above in the categorization by Barley et al. [74], women from different occupational sectors, women from different countries, and so on). We argue that more insight into these intra- and inter-individual changes will shed more light to the question how menopause can affect the career sustainability over time, and possible negative effects can be combatted. 

Last but not least, it might also be beneficial to conduct future research adopting an even broader approach wherein scholarly work from both the medical and work/career fields are combined, to more elaborately study the impact of menopause on women’s work and private life, using an intersectional lens [79]. Obviously, for this research line to work well, it is important to include researchers from both fields in the author team in order to safeguard the validity of the selection criteria. 

In order to do so, longitudinal research designs using several measurement moments, and ideally incorporating multi-source ratings [80] from multiple stakeholders, are needed in order to detect developmental patterns [81] and to understand sequences of causes and effects [82]. Obviously, given the complexity of understanding the phenomenon of menopause at the workplace from a sustainable career perspective, qualitative work is also needed to gain in-depth knowledge on women’s perceptions about their career sustainability in relation to menopause, what influences those, the impact of stakeholders in this regard, and how these perceptions evolve over time. Last but not least, more scholarly work is needed to determine how one’s supervisor and organizational support systems might help and be optimized in order to sustain menopausal women at the workplace. 

Obviously, more knowledge on the impact of menopause on sustainable career outcomes may guide management and HRM practitioners in dealing with both negative and positive experiences women, and their surrounding stakeholders, face when going through menopause. Hopefully, more insight into the phenomenon will also help to combat (meta)stereotyping (e.g., [83]) around menopause, to facilitate the career sustainability of women in the second half of their career. From a sustainable career perspective, organizational interventions need to address employees’ current health, happiness and productivity (being the core indicators of sustainable careers) while taking into account their future health, happiness and productivity (see also [84]). Sustainability of women’s careers in the second half of life is of increasing importance given the increasing equal representation of men and women in working organizations [13], and the impact of the changing nature of work in the 21st century on older workers [85]. 

Following the work by Nelson and associates [86], who searched for evidence-based practice regarding the management of menopause-related symptoms, we support the adoption of organizational interventions aimed at raising the awareness of healthy dietary behavior requirements and the need for physical exercise. Management in organizations could also support women suffering from complaints by offering manual and/or energy therapies or psychosocial counseling at work, herewith facilitating their time management, or by means of financial support to attend these (outside working hours). 

Just like all studies, this contribution has some limitations. First of all, as our research approach only considered documents from the WoS (being the world’s leading academic database in social science), in particular from the domains of Public Environmental Occupational Health, Behavioral Sciences, Business, Management, Psychology Applied, Ergonomics, Psychology, Psychology Multidisciplinary, and Social Sciences Interdisciplinary, we might have missed some interesting work. Nevertheless, we argue that the scholarly fields that we included in our bibliometric analysis comprise the main literature regarding the phenomenon of menopause at the workplace. Moreover, although the document selection was grounded on a carefully composed list of WoS keywords, we still might have missed some documents in case the author(s) did not mention relevant keywords (in fact, the older documents do not include keywords), and/or there may have been some bias in the use of keywords. Notwithstanding these limitations, a strong point of SciMAT, the tool that we have used in our work, is that it allowed us to refine the keywords in order to obtain more accurate results. 

## Figures and Tables

**Table 1:** String used to search in the WoS.

TS = ((Job* or career* or employment* or employee* or professional* or “work-related health” or “work ability” or “ability to work” or “work outcome*” or “experience of work” or burnout or vitality or “work engagement” or “motivation at work” or “motivation to work” or “motivation to continue working” or “work centrality” or “job satisfaction” or “job performance” or employability) and (menopau* or climacteric* or amenorrhoea or amenorroe))

* after a keyword in the search criteria means that the system will search for all possible keywords for this root, that is, plurals, etc. (e.g., work* will search for worker, workers, etc.).

**Table 2:** Most productive authors in the menopause and sustainable careers outcomes scientific domain.

Author	Documents
Raczkiewicz, D.; Bojar, I.	4
Kingsberg, S.A.; Simon, J.A.; Hirokawa, K.; Humeniuk, E.; Grochans, E.; Szkup, M.; Karakiewicz, B.; Jurczak, A.; Lope, V.; Garcia-Perez, J.; Perez-Gomez, B.; Angel Alba, M.; van der Haar, Rudolf; Pedraz-Pingarron, C.; Moreo, P.; Santamarina, C.; Ederra, M.; Salas-Trejo, D.; Sanchez-Contador, C.; Llobet, R.; Pollan, M.; Owoc, A.; Ghorbani, R.; Nassaji, M.; Shahbazi, A.; Bien, A.; Rutanen, R.; Luoto, R.; Tomas, E.; Nygard, C.; Willett, W.C.; Boughton, M.; Brown, D.E.; Brown, D.E; Ariyoshi, H.; Gold, EB; Derry, PS; Mansfield, PK; Koch, PB; Matthews, KA; Bromberger, J; Wilbur, J	2

**Table 3:** Highest cited publications and authors in the menopause and sustainable careers outcomes scientific domain.

	Title	Author/s	Year	Citations
1	Factors associated with age at natural menopause in a multiethnic sample of midlife women	Gold, EB, Bromberger, J, Crawford, S, Samuels, S, Greendale, GA, Harlow, SD, Skurnick, J	2001	488
2	Then and now: Quality of life of young breast cancer survivors	Bloom, JR, Stewart, SL, Chang, S, Banks, PJ	2004	259
3	The influence of age and sex on the prevalence of depressive conditions: report from the National Survey of Psychiatric Morbidity	Bebbington, PE, Dunn, G, Jenkins, R, Lewis, G, Brugha, T, Farrell, M, Meltzer, H	1998	217
4	Physical and psychosocial problems in cancer survivors beyond return to work: a systematic review	Duijts, SFA, Van Egmond, MP, Spelten, E, Van Muijen, P, Anema, JR, Van der Beek, AJ	2014	139
5	Health-related quality of life in a multiethnic sample of middle-aged women—Study of Women’s Health Across the Nation (SWAN)	Avis, NE, Ory, M, Matthews, KA, Schocken, M, Bromberger, J, Colvin, A	2003	115
6	Reproductive history and mortality in late middle age among Norwegian men and women	Grundy, E, Kravdal, O	2008	96
7	gender inequalities in health— social position, affective-disorders and minor physical morbidity	Popay, J, Bartley, M, Owen, C	1993	91
8	Mortality from diseases of the circulatory system in radiologic technologists in the United States	Hauptmann, M, Mohan, AK, Doody, MM, Linet, MS, Mabuchi, K	2003	62
9	Decision-making and hormone replacement therapy: A qualitative analysis	Hunter, MS, ODea, I, Britten, N	1997	62
10	Impact of occupational exposure on lead levels in women	Popovic, M, McNeill, FE, Chettle, DR, Webber, CE, Lee, CV, Kaye, WE	2005	59
11	Evaluation of a group cognitive behavioural intervention for women suffering from menopausal symptoms following breast cancer treatment	Hunter, MS, Coventry, S, Hamed, H, Fentiman, I, Grunfeld, EA	2009	58
12	A survey on issues in the lives of women with severe mental illness	Ritsher, JEB, Coursey, RD, Farrell, EW	1997	58
13	Economic burden of osteoporosis, breast cancer, and cardiovascular disease among postmenopausal women in an employed population	Sasser, AC, Rousculp, MD, Birnbaum, HG, Oster, EF, Lufkin, E, Mallet, D	2005	57
14	Lactation in relation to postmenopausal breast cancer	Newcomb, PA, Egan, KM, Titus-Ernstoff, L, Trentham-Dietz, A, Greenberg, ER, Baron, JA, Willett, WC, Stampfer, MJ	1999	55

**Table 4:** Most prolific journals (articles and reviews) in the menopause and sustainable careers outcomes scientific domain.

	Journals	Documents
1	BMC WOMENS HEALTH	10
2	INTERNATIONAL JOURNAL OF ENVIRONMENTAL RESEARCH AND PUBLIC HEALTH	10
3	JOURNAL OF WOMENS HEALTH	10
4	AMERICAN JOURNAL OF EPIDEMIOLOGY	8
5	WOMENS HEALTH ISSUES	7
6	WOMEN & HEALTH	7
7	SOCIAL SCIENCE & MEDICINE	6
8	ANNALS OF AGRICULTURAL AND ENVIRONMENTAL MEDICINE	5
9	HEALTH CARE FOR WOMEN INTERNATIONAL	5
10	JOURNAL OF OCCUPATIONAL AND ENVIRONMENTAL MEDICINE	5
11	BMC PUBLIC HEALTH	4
12	WORK-A JOURNAL OF PREVENTION ASSESSMENT & REHABILITATION	4
13	PSYCHO-ONCOLOGY	4
14	JOURNAL OF WOMENS HEALTH & GENDER-BASED MEDICINE	4

## Data Availability

Not applicable.

## References

[1] Okeke T.C., Ezenyeaku C.C.T., Ikeako L.G., Agu P.U. (2013). An overview of menopause associated Vaso Motor Symptoms and options available in its management. Niger. J. Med..

[2] Atkinson C., Beck V., Brewis J., Davies A., Duberley J. (2020). Menopause and the Workplace: New Directions in HRM Research and HR Practice. Hum. Resour. Manag. J..

[3] Thomas F., Renaud F., Beneiace E., De Mfeus T. (2001). International Variability of Ages at Menarche and Meno-Pause: Patterns and Main Determinants. Hum. Biol..

[4] Grandey A.A., Gabriel A.S., King E.B. (2020). Tackling Taboo Topics: A Review of the Three M s in Working Women’s Lives. J. Manag..

[5] Bariola E., Jack G., Pitts M., Riach K., Sarrel P. (2017). Employment Conditions and Work-Related Stressors are Associated with Menopausal Symptom Reporting among Perimenopausal and Postmenopausal Women. Menopause.

[6] Hickey M., Riach K., Kachouie R., Jack G. (2017). No Sweat: Managing Menopausal Symptoms at Work. J. Psychosom. Obstet. Gynecol..

[7] Griffiths A., MacLennan S.J., Hassard J. (2013). Menopause and Work: An Electronic Survey of Employees’ Attitudes in the UK. Maturitas.

[8] High R.V., Marcellino P.A. (1994). Menopausal Women and the Work Environment. Soc. Behav. Personal. Int. J..

[9] Steffan B. (2020). Managing Menopause at Work: The Contradictory Nature of Identity Talk. Gend. Work. Organ..

[10] De Vos A., Van der Heijden B.I.J.M., Akkermans J. (2020). Sustainable Careers: Towards a Conceptual Model. J. Vocat. Behav..

[11] Van der Heijden B., De Vos A., Akkermans J., Spurk D., Semeijn J., Van der Velde M., Fugate M. (2020). Special Issue Sustainable Careers across the Lifespan: Moving the Field Forward. Introductory Article. J. Vocat. Behav..

[12] Van der Heijden B.I.J.M., De Vos A. (2015). Sustainable Careers: Introductory Chapter. Handbook of Research on Sustainable Careers.

[13] Catalyst Quick Take: Women in the workforce—Global (October 31) 2018. http://www.catalyst.org/research/.

[14] Jack G., Riach K., Bariola E. (2019). Temporality and gendered agency: Menopausal subjectivities in women’s work. Hum. Relat..

[15] Akinola M. (2010). Measuring the Pulse of an Organization: Integrating Physiological Measures into the Organizational Scholar’s Toolbox. Res. Organ. Behav..

[16] Ganster D.C., Rosen C.C. (2013). Work Stress and Employee Health: A Multidisciplinary Review. J. Manag..

[17] Van der Heijden B.I.J.M. (2005). “No One Has Ever Promised You a Rose Garden” on Shared Responsibility and Employability En-Hancing Strategies throughout Careers.

[18] Marcus-Newhall A., Thompson S., Thomas C. (2001). Examining a Gender Stereotype: Menopausal Women. J. Appl. Soc. Psychol..

[19] Winakur J. (2011). ‘Frankenfolks’ and the Rise of Ageism. Health Aff..

[20] Jack G., Riach K., Bariola E., Pitts M., Schapper J., Sarrel P. (2016). Menopause in the Workplace: What Employers Should Be Doing. Maturitas.

[21] Mvundura V. (2007). Menopause Transition and Labor Market Outcomes. Dissertation Georgia State University. http://scholarworks.gsu.edu/econ_diss/38.

[22] Monteleone P., Mascagni G., Giannini A., Genazzani A.R., Simoncini T. (2018). Symptoms of Menopause—Global Prevalence, Physiology and Implications. Nat. Rev. Endocrinol..

[23] Bromberger J.T., Kravitz H.M., Chang Y.F., Cyranowski J.M., Brown C., Matthews K.A. (2011). Major depression during and after the menopausal transition: Study of Women’s Health Across the Nation (SWAN). Psychol. Med..

[24] Small H. (1999). Visualizing Science by Citation Mapping for Information Science. J. Am. Soc..

[25] Cobo M., López-Herrera A., Herrera-Viedma E., Herrera F. (2011). Science Mapping Software Tools: Review, Analysis, and Cooperative Study Among Tools. J. Am. Soc. Inf. Sci. Technol..

[26] Zupic I., Čater T. (2014). Bibliometric Methods in Management and Organization. Organ. Res. Methods.

[27] Cobo M.J., López-Herrera A.G., Herrera-Viedma E., Herrera F. (2012). SciMAT: A New Science Mapping Analysis Software Tool. J. Am. Soc. Inf. Sci. Technol..

[28] Colakoglu S., Lepak D.P., Hong Y. (2006). Measuring HRM Effectiveness: Considering Multiple Stakeholders in a Global Context. Hum. Resour. Manag. Rev..

[29] Johns G. (2006). The Essential Impact of Context on Organizational Behavior. Acad. Manag. Rev..

[30] Rostosky S.S., Travis C.B. (1996). Menopause Research and The Dominance of the Biomedical Model 1984–1994. Psychol. Women Q..

[31] Hirschi A., Steiner R., Burmeister A., Johnston C.S. (2020). A Whole-Life Perspective of Sustainable Careers: The Nature and Consequences of Nonwork Orientations. J. Vocat. Behav..

[32] Aguinis H., Pierce C.A., Bosco F.A., Muslin I.S. (2009). First Decade of Organizational Research Methods: Trends in Design, Measurement, and Data-Analysis Topics. Organ. Res. Methods.

[33] Hood W.W., Wilson C.S. (2001). The Literature of Bibliometrics, Scientometrics, and Informetrics. Scientometrics.

[34] Pritchard A. (1969). Statistical Bibliography or Bibliometrics. J. Doc..

[35] Garfield E. (1979). Is Citation Analysis a Legitimate Evaluation Tool?. Scientometrics.

[36] Rosenthal R. (1979). The File Drawer Problem and Tolerance for Null Results. Psychol. Bull..

[37] Moral-Muñoz J.A., Herrera-Viedma E., Santisteban-Espejo A., Cobo M.J. (2020). Software Tools for Conducting Bibliometric Analysis in Science: An Up-To-Date Review. Prof. Inf..

[38] Börner K., Chen C., Boyack K.W. (2005). Visualizing Knowledge Domains. Annu. Rev. Inf. Sci. Technol..

[39] Falagas M.E., Pitsouni E.I., Malietzis G.A., Pappas G. (2008). Comparison of PubMed, Scopus, Web of Science, and Google Scholar: Strengths and Weaknesses. FASEB J..

[40] Martínez M.A., Cobo M.J., Herrera M., Herrera-Viedma E. (2015). Analyzing the Scientific Evolution of Social Work Using Science Mapping. Res. Soc. Work Pract..

[41] Callon M., Courtial J.-P., Turner W.A., Bauin S. (1983). From Translations to Problematic Networks: An Introduction to Co-Word Analysis. Soc. Sci. Inf..

[42] Coulter N., Monarch I., Konda S. (1998). Software Engineering as Seen Through its Research Literature: A Study in Co-Word Analysis. J. Am. Soc. Inf. Sci..

[43] Callon M., Courtial J.P., Laville F. (1991). Co-Word Analysis as a Tool for Describing the Network of Interactions Between Basic and Technological Research: The Case of Polymer Chemsitry. Scientometrics.

[44] Gold E.B., Bromberger J., Crawford S., Samuels S., Greendale G.A., Harlow S.D., Skurnick J. (2001). Factors Associated with Age at Natural Menopause in a Multiethnic Sample of Midlife Women. Am. J. Epidemiol..

[45] Duijts S.F.A., Van Egmond M.P., Spelten E., Van Muijen P., Anema J.R., Van Der Beek A.J. (2014). Physical and Psychosocial Problems in Cancer Survivors Beyond Return to Work: A Systematic Review. Psycho-Oncology.

[46] Bebbington P.E., Dunn G., Jenkins R., Lewis G., Brugha T., Farrell M., Meltzer H. (1998). The Influence of Age and Sex on the Prevalence of Depressive Conditions: Report from the National Survey of Psychiatric Morbidity. Psychol. Med..

[47] Chirawatkul S., Manderson L. (1994). Perceptions of Menopause in Northeast Thailand: Contested Meaning and Practice. Soc. Sci. Med..

[48] Green E.E., Thompson D., Griffiths F. (2002). Narratives of Risk: Women at Midlife, Medical ‘experts’ and Health Technologies. Health Risk Soc..

[49] Hunter M.S., O’Dea I., Britten N. (1997). Decision-Making and Hormone Replacement Therapy: A Qualitative Analysis. Soc. Sci. Med..

[50] Davis M.C., Matthews K.A., Meilahn E.N., Kiss J.E. (1995). Are Job Characteristics Related to Fibrinogen Levels in Middle-Aged Women?. Health Psychol..

[51] Wamala S.P., Wolk A., Schenck-Gustafsson K., Orth-Gomer K. (1997). Lipid Profile and Socioeconomic Status in Healthy Middle Aged Women in Sweden. J. Epidemiol. Community Health.

[52] Gardner K.M., Shu X.O., Jin F., Dai Q., Ruan Z., Thompson S.J., Hussey J.R., Gao Y.T., Zheng W. (2002). Occupations and Breast Cancer Risk among Chinese Women in Urban Shanghai. Am. J. Ind. Med..

[53] Kleinman N.L., Rohrbacker N.J., Bushmakin A.G., Whiteley J., Lynch W.D., Shah S.N. (2013). Direct and Indirect Costs of Women Diagnosed with Menopause Symptoms. J. Occup. Environ. Med..

[54] Popovic M., McNeill F.E., Chettle D.R., Webber C.E., Lee C.V., Kaye W.E. (2005). Impact of Occupational Exposure on Lead Levels in Women. Environ. Health Perspect..

[55] Bloom J.R., Stewart S., Chang S., Banks P.J. (2004). Then and Now: Quality of Life of Young Breast Cancer Survivors. Psycho-Oncology.

[56] Outram S., Mishra G.D., Schofield M.J. (2004). Sociodemographic and Health Related Factors Associated with Poor Mental Health in midlife Australian Women. Women Health.

[57] Cassou B., Mandereau L., Aegerter P., Touranchet A., Derriennic F. (2007). Work-related Factors Associated with Age at Natural Menopause in a Generation of French Gainfully Employed Women. Am. J. Epidemiol..

[58] Fallahzadeh H. (2010). Quality of Life after the Menopause in Iran: A Population Study. Qual. Life Res..

[59] Sasser A.C., Rousculp M.D., Birnbaum H.G., Oster E.F., Lufkin E., Mallet D. (2005). Economic Burden of Osteoporosis, Breast Cancer, and Cardiovascular Disease among Postmenopausal Women in an Employed Population. Women’s Health Issues.

[60] Hauptmann M., Mohan A.K., Doody M.M., Linet M.S., Mabuchi K. (2003). Mortality from Diseases of the Circulatory System in Radiologic Technologists in the United States. Am. J. Epidemiol..

[61] Wang X.-S., Travis R.C., Reeves G., Green J., Allen E.N., Key T.J., Roddam A.W., Beral V. (2012). Characteristics of the Million Women Study Participants Who Have and Have Not Worked at Night. Scand. J. Work. Environ. Health.

[62] Bien A., Rzonca E., Iwanowicz-Palus G., Pańczyk-Szeptuch M. (2015). The Influence of Climacteric Symptoms on Women’s Lives and Activities. Int. J. Environ. Res. Public Health.

[63] Ghorbani R., Nassaji M., Shahbazi A., Rostami B., Taheri M. (2015). Association between Quality of Life, Menopausal Status, and Sociodemographic Factors among Middle-Aged Women in Iran. J. Egypt. Public Health Assoc..

[64] Converso D., Viotti S., Sottimano I., Loera B., Molinengo G., Guidetti G. (2019). The Relationship between Menopausal Symptoms and Burnout. A Cross-Sectional Study among Nurses. BMC Women’s Health.

[65] Raczkiewicz D., Owoc A., Sarecka-Hujar B., Saran T., Bojar I. (2017). Impact of Spinal Pain on Daily Living Activities in Post-Menopausal Women Working in Agriculture. Ann. Agric. Environ. Med..

[66] Rutanen R., Nygård C.-H., Moilanen J., Mikkola T., Raitanen J., Tomas E., Luoto R. (2014). Effect of Physical Exercise on Work Ability and Daily Strain in Symptomatic Menopausal Women: A Randomized Controlled Trial. Work.

[67] Verburgh M., Verdonk P., Appelman Y., Zanten M.B.-V., Nieuwenhuijsen K. (2020). “I Get That Spirit in Me”—Mentally Empowering Workplace Health Promotion for Female Workers in Low-Paid Jobs During Menopause and Midlife. Int. J. Environ. Res. Public Health.

[68] Wieder-Huszla S., Szkup M., Jurczak A., Samochowiec A., Samochowiec J., Stanisławska M., Rotter I., Karakiewicz B., Grochans E. (2014). Effects of Socio-Demographic, Personality and Medical Factors on Quality of Life of Postmenopausal Women. Int. J. Environ. Res. Public Health.

[69] Abdelrahman R.Y., Abushaikha L.A., al-Motlaq M.A. (2014). Predictors of Psychological Well-Being and Stress among Jordanian Menopausal Women. Qual. Life Res..

[70] Watkins E.R., Walker A., Mol E., Jahnke S., Richardson A.J. (2019). Women Firefighters’ Health and Well-Being: An International Survey. Women’s Health Issues.

[71] Grochans E., Szkup M., Kotwas A., Kopeć J., Karakiewicz B., Jurczak A. (2018). Analysis of Sociodemographic, Psychological, and Genetic Factors Contributing to Depressive Symptoms in Pre-, Peri-and Postmenopausal Women. Int. J. Environ. Res. Public Health.

[72] Moen P. (1996). A Life Course Perspective on Retirement, Gender, and Well-Being. J. Occup. Health Psychol..

[73] Roberts B.W., Friend W. (1998). Career Momentum in Midlife Women: Life Context, Identity, and Personality Correlates. J. Occup. Health Psychol..

[74] Barley S.R., Bechky B.A., Milliken F.J. (2017). The Changing Nature of Work: Careers, Identities, and Work Lives in the 21stCentury. Acad. Manag. Discov..

[75] Kooij D.T.A.M., Jansen P.G.W., Dikkers J.S.E., De Lange A.H. (2010). The Influence of Age on the Associations between HR Practices and both Affective Commitment and Job Satisfaction: A Meta-Analysis. J. Organ. Behav..

[76] Veth K.N., Korzilius H.P.L.M., Van Der Heijden B.I.J.M., Emans B.J.M., De Lange A.H. (2019). Which HRM Practices Enhance Employee Outcomes at Work Across the Life-Span?. Int. J. Hum. Resour. Manag..

[77] De Jonge J., Dormann C. (2006). Stressors, Resources, and Strain at Work: A Longitudinal Test of the Triple-Match Principle. J. Appl. Psychol..

[78] Martin M., Hofer S.M. (2004). Intraindividual Variability, Change, and Aging: Conceptual and Analytical Issues. Gerontology.

[79] Cole E.R. (2009). Intersectionality and Research in Psychology. Am. Psychol..

[80] Podsakoff P.M., MacKenzie S.B., Lee J.Y., Podsakoff N.P. (2003). Common Method Biases in Behavioral Research: A Critical Review of the Literature and Recommended Remedies. J. Appl. Psychol..

[81] Laberge M., Ledoux E. (2011). Occupational Health and Safety Issues Affecting Young Workers: A Literature Review. Work.

[82] Frese M., Zapf D., Cooper C.L., Payne R. (1988). Methodological Issues in the Study of Work Stress: Objective vs Subjective Measurement of Work Stress and the Question of Longitudinal Studies. Causes, Coping and Consequences of Stress at Work.

[83] Peters P., Van der Heijden B.I.J.M., Spurk D., De Vos A., Klaassen R. (2019). Please Don’t Look at Me That Way. An Empirical Study into the Effects of Age-Based (Meta-) Stereotyping on Employability Enhancement among Older Supermarket Workers. Front. Psychol..

[84] Van Engen M.L., Vinkenburg C., Dikkers J.S.E. (2012). Sustainability in Combining Career and Care: Challenging Normative Beliefs about Parenting. J. Soc. Issues.

[85] Ackerman P.L., Kanfer R. (2020). Work in the 21st Century: New Directions for Aging and Adult Development. Am. Psychol..

[86] Nelson H.D., Haney E., Humphrey L., Miller J., Nedrow A., Nicolaidis C., Vesco K., Walker M., Bougatsos C., Nygren P. (2005). Management of Menopause-Related Symptoms. Summary, Evidence Report/Technology Assessment No. 120.

